# Clinical adoption of robotics in endoscopy: Challenges and solutions

**DOI:** 10.1002/jgh3.12412

**Published:** 2020-09-09

**Authors:** Hung Leng Kaan, Khek Yu Ho

**Affiliations:** ^1^ Department of General Surgery Ng Teng Fong General Hospital Singapore; ^2^ Department of General Surgery National University Hospital Singapore; ^3^ Department of Surgery, Yong Loo Lin School of Medicine National University of Singapore Singapore; ^4^ Department of Medicine, Yong Loo Lin School of Medicine National University of Singapore Singapore

**Keywords:** flexible endoscopy, robotics, therapeutic endoscopy

## Abstract

The endoscope was traditionally used as a diagnostic instrument. In past decades, it has increasingly been adapted for therapeutic intents. Subsequently, the master–slave robotic concept was introduced into the field of endoscopy to potentially reduce the difficulty and complication rates of endoscopic therapeutic procedures. As interest in robotic endoscopy intensified, progressively more robotic endoscopic platforms were developed, tested, and introduced. Nevertheless, the future of robotic endoscopy hinges on the ability to meet specific clinical needs of procedurists. Three aspects are vital in ensuring continued success and clinical adoption of the robotic endoscope—demonstration of clinical safety and cost‐efficacy of the device, widespread availability of directed training opportunities to enhance technical skills and clinical decision‐making capabilities of the procedurist, and continued identification of new clinical applications beyond the current uses of the device. This review provides a brief discussion of the historical development of robotic endoscopy, current robotic endoscopic platforms, use of robotic endoscopy in conventional therapeutic endoscopic procedures, and the future of robotic endoscopy.

## Graphical abstract

A successful robotic endoscope must provide beyond the basic requirement of a high functioning endoscopic resection tool. It must be demonstrated to be safe, and cost‐effective for its intended clinical application. Training can be best achieved through the synergy of artificial‐intelligence‐guided deep learning systems and virtual reality. Natural Orifice Transluminal Endoscopic Surgery (NOTES) is an ideal choice for the application of the robotic endoscope as the triangulation afforded by the device effectively solves the challenges faced when performing NOTES using existing endoscopic equipment.

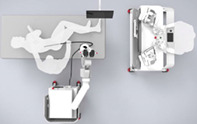

## Introduction

Conventional endoscopic instruments present numerous challenges to users performing complex endoscopic procedures. The biggest obstacle is the lack of triangulation, spatial orientation, and optimal tissue retraction. This creates a nearly insurmountable obstacle should procedurists wish to perform basic surgical techniques, such as suturing, retraction, and ligation, using the endoscope.^1^

With the adaption of the master–slave robotic concept to the endoscope, new robotic endoscopic platforms offer exciting new opportunities for endoscopists to perform complex endoscopic procedures with less difficulty and reduced complication rates.

Nevertheless, for robotic endoscopes to successfully gain widespread clinical adoption, and complement or even replace existing gold standard endoscopic platforms, three enabling milestones must be achieved—establishment of clinical safety, efficacy, and cost‐effectiveness; well‐designed training opportunities for users; and continued identification of new clinical applications for the device.

First, clinical safety and effectiveness of the robotic endoscope must be demonstrated via high quality large‐scale human clinical trials. Important clinical variables to identify during the conduct of the human trial include safety endpoints such as major adverse events, and performance endpoints such as technical success of the procedure. At the same time, health technology assessment should also be conducted, where value driven endpoints such as cost‐effectiveness of the novel technology are measured against the existing gold standards.

Second, training opportunities adapted to meet the specific needs of procedurists must be readily available. In the fields of endoscopy and robotic surgery, virtual reality (VR) training solutions have been demonstrated to be efficacious, convenient, and cost effective.^2^, ^3^ Nevertheless, although VR training solutions can help to improve the technical skills of the procedurist, it does not enhance the procedurist's clinical decision‐making process as there is a lack of real‐time performance feedback to the user. This challenge can potentially be overcome by adapting an artificial‐intelligence‐based deep learning system to the VR training solution for the robotic endoscope.

Third, continued new clinical applications must be identified to enhance value and impact of the robotic endoscope. One such application is the adaptation of the robotic endoscope to Natural Orifice Transluminal Endoscopic Surgery (NOTES). Because of the primary benefit of triangulation offered by the robotic endoscope, it lends itself as an excellent tool for surmounting the obstacles currently faced when performing NOTES.

## Historical development of robotic endoscopy

Endoscopy began as a method for diagnosing pathologies in the gastrointestinal tract. Over the decades, endoscopes have evolved, allowing procedurists to perform a multitude of tasks—biopsies, hemostasis, polypectomies, complex therapeutic endoscopic resections such as endoscopic mucosal resections (EMR) and endoscopic submucosal dissections (ESD), and even peroral surgical procedures such as peroral endoscopic myotomy (POEM).

Nevertheless, traditional endoscopic instruments have very limited degrees of freedom, making it extremely challenging for even experienced procedurists to perform complex endoscopic procedures. As a result, only selected cases could be performed endoscopically, and if complications were encountered, they could only be solved using surgical methods.^4^ The wish was therefore for a new and improved version of the endoscope that could overcome these problems and provide the benefits of surgery—triangulation, adequate tissue retraction, and optimal exposure of the operating field.

Although robots are commonly used in the industry, it was only recently that robots made their entry into the medical field. In the 2000s, the concept of master–slave robots was introduced into the field of minimally invasive surgery. The da Vinci and ZEUS platforms were the forerunners of the surgical master–slave systems. Eventually, the da Vinci platform dominated over the ZEUS platform because of its surgical instruments that allowed articulation at the wrist to seven degrees of freedom, effectively mimicking the functions and movements of the human wrist.^5^

This unique feature of the robot perfectly lent itself to overcoming the challenges faced in conventional endoscopy. Before long, the master–slave robotic concept was applied to the field of endoscopy, and this then led to the development of robotic endoscopic platforms. With the new robotic endoscopic platforms, the hope is that procedurists can better perform complex endoscopic therapeutic procedures and surgeons can perform NOTES.

## Robotic endoscopic platforms

Most of the existing robotic endoscopic platforms consist of a flexible robotic endoscope that can be telemanipulated. Within the robotic endoscope, there are usually at least two articulating end‐effectors capable of achieving triangulation, adequate tissue retraction, and optimal exposure of the operating field. In this review, we will discuss the Endomaster Endoluminal Access Surgical Efficacy (EASE) System and the ISIS‐Scope/STRAS System.

### *Endomaster EASE System*

**Figure 1:**
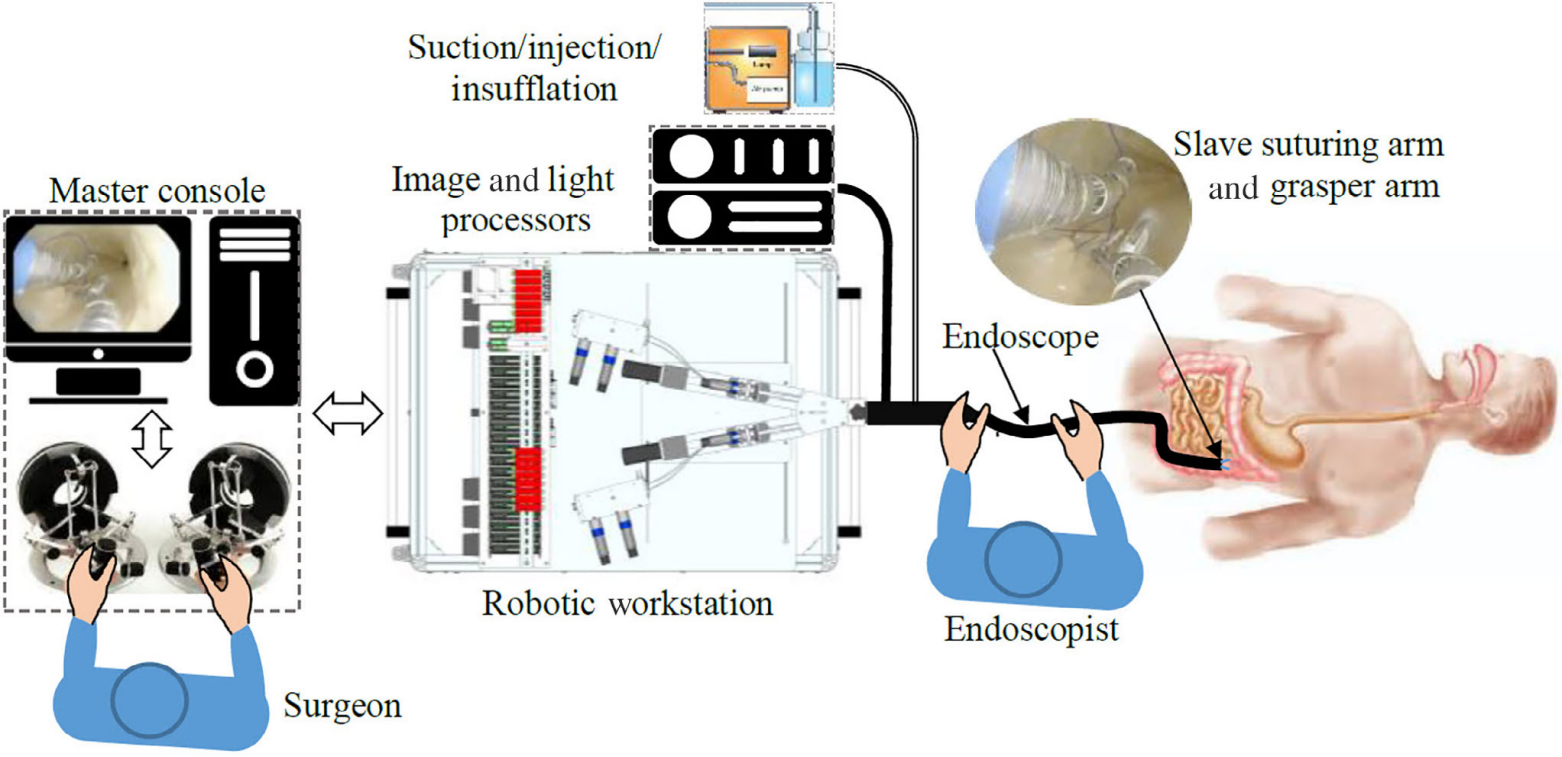
Set up of the Endomaster Endoluminal Access Surgical Efficacy System.

The Endomaster EASE System (Endomaster Pte Ltd, Singapore), previously known as Master and Slave TransEndoluminal Robot (MASTER), is a flexible robotic endoscopic platform. The platform comprises of a master console, a telesurgical work station, and a robotic endoscope (Fig. 1). The robotic endoscope has three instrument channels. One channel is found within the core of the scope's shaft for insertion of conventional non‐robotic endoscopic instruments. The other two instrument channels allow robotic instruments to be inserted. The robotic arms have nine degrees of freedom and can be telemanipulated. The robotic instruments are interchangeable and include grasper, hook, and needle holder for suturing.^6^ The robotic needle holder is a modified grasper with two needle holes that can lock and release a double‐pointed lancet needle attached to a suture.^7^

### *ISIS‐Scope/STRAS System*

The ISIS‐Scope/STRAS System (Karl Storz, IRCAD, Tuttlingen, Germany) is a flexible robotic system based on a modified shortened version of the manual Anubiscope. The robotic endoscope has three instrument channels. One channel is found within the core of the scope's shaft, and the other two lateral channels are located at the edges of the shaft within mobile shells. The two lateral instruments can deviate from the main direction of the endoscope when the mobile shells are opened. The endoscopic instruments have hollow shafts. Inserts equipped with mechanical or electrical effectors can be attached to the instruments by screwing the inserts onto the distal end of the hollow shafts. These characteristics allow the ISIS‐Scope/STRAS System to have ten degrees of freedom.^8^

## Use of robotic endoscopy in conventional therapeutic endoscopic procedures and its benefits

### *Reduces difficulty of performing complex therapeutic procedures*

The two robotic arms of the Endomaster EASE System effectively mimics the wrists of a surgeon. Because of its high degrees of freedom, the procedurist can achieve triangulation and obtain adequate tissue retraction with optimal exposure of the operating field. The end‐effector telemanipulation features also allow users to focus their efforts on performing the task at hand, instead of being involved in tedious repetitive manual tasks required in traditional endoscopy.^9^ These advantages are especially crucial in reducing the complication rates and time taken to perform complex therapeutic endoscopic procedures.

In our prospective human case series, we demonstrated that the mean procedural duration for performing ESD with the Endomaster EASE System was 39 min (26–68 min). The average size of the gastric lesions was 2.2 cm (1.5–3 cm). For all five patients enrolled in this human trial, complete resection of the gastric neoplasms was achieved with no complications.^10^

This is in stark contrast to the average time required to perform ESD using conventional endoscopy. Fujimoto *et al*. analyzed 18 cases of ESD performed on early gastric cancer. The average procedural time was 87.0  ±  43.1 min and the average length of the longer axis of the lesions was 2.74 ±  1.00 cm. For adverse events, the perforation rate and the delayed bleeding rate were both 5.5%.^11^

For colonic ESD, Turiani Hourneaux de Moura *et al*. also demonstrated that the total procedure time was lower when using robotic endoscopy as compared to using conventional endoscopy (34.1 *vs* 88.6 min, *P* = 0.001). The perforation rate was also higher when using conventional endoscopy (60 *vs* 30%, *P* = 0.18).^12^

### *Ability to manage complications endoscopically*

The two most common complications encountered in performing ESD is perforation and bleeding. The suturing device of the Endomaster EASE System provides a unique solution to both complications. The ability to achieve triangulation with the two robotic arms is fundamental in performing effective suturing. In addition, the suturing device allows for secure surgical knot tying. This is in contrast to currently available endoscopic suturing devices, such as OverStitch, which relies on fastening elements.^7^

In our animal trial, we used the Endomaster EASE System to perform a 10 mm submucosal incision in the colon. We then used the robotic needle holder and robotic grasper to apply a Figure‐of‐8 stitch. Subsequently, we tied surgical knots to close the perforation. The process of suturing the Figure‐of‐8 stitch using the robotic suturing endoscopic device required 11 min. The process of knot tying required 4 min.^7^, ^9^

## The future of robotic endoscopy

### *Establishing clinical safety and efficacy of the robotic endoscope*

Till date, the Endomaster EASE System has been clinically validated in animal and small‐scale human trials for ESD of early gastric neoplasia.^10^, ^13^, ^14^ User interactions with the system were also studied comparing the performances of novices and experienced endoscopists on the Endomaster EASE System. With the Endomaster EASE System, novices without endoscopy experience could complete the ESD procedure.^15^ Moving forward, the Endomaster EASE System will be evaluated for the treatment of colorectal neoplasms that cannot be optimally and completely resected using existing endoscopic snare‐based techniques.

### *Optimizing training solutions for the robotic endoscope using an artificial‐intelligence‐based deep learning system adapted to a VR training simulation*

Despite best efforts to demonstrate the safety and efficacy of the robotic endoscope, it can only be effectively adopted in clinical practice if there exists an appropriately designed training tool to meet the needs of its new users. To this end, we believe that an artificial‐intelligence‐based deep learning system adapted to a VR training simulation would be the most ideal and valuable training tool for procedurists.

VR training tools have already been shown to be effective in endoscopy and robotic surgery training. Hence, we believe that VR is the optimal platform for training users of robotic endoscopy. A Cochrane review and meta‐analysis performed by Khan *et al*. evaluated the role of VR simulation training in endoscopy.^2^ Khan *et al*. showed that VR simulation is advantageous over no training and can be used to supplement conventional endoscopic training. In the field of training for robotic surgery, Bric *et al*. identified several advantages of VR training such as safety, efficacy in acquiring robotic surgical skills, cost effectiveness, and convenience.^3^

However, a big drawback of VR as a standalone training solution is that it is limited to enhancing users' technical abilities and does not promote real‐time performance feedback. With the use of artificial‐intelligence‐guided deep learning analytical methods, on‐the‐spot advice can be provided to users to enhance their learning experiences, and in the future, clinical decision‐making capabilities.

Currently, there exists a multitude of methods that can be used to develop deep learning systems. The most commonly used method for image and video analysis is based on convolutional neural networks (CNNs). CNNs perform similarly to a network of neurons in the human brain. To develop a high quality CNN, clean and complete data must be available. This can be done by collating a large database, hence allowing for sufficient amount of data to be available for machine deep learning. Images and videos must also be accurately annotated to create a valuable and useful database.^16^

Hirasawa *et al*. used artificial intelligence with deep learning to develop CNNs which could automatically detect gastric cancer in endoscopic images.^17^ The CNN‐based diagnostic system was trained using more than 13 500 endoscopic images of gastric cancer. Overall, the diagnostic system had a sensitivity of 92.2%. Everson *et al*. trained CNNs in detecting and classifying early esophageal squamous cell carcinoma.^18^ With an accuracy of 93.7% and a sensitivity of 89.3%, this diagnostic system was able to operate in real‐time and predict a diagnosis between 26.17 ms and 37.48 ms.

### *Identifying new clinical applications for the robotic endoscope*

Finally, for the robotic endoscope to be adopted by mainstream clinical practice, its continued relevance to practitioners must be ensured. To achieve this aim, it is paramount that new clinical applications for the robotic endoscope are continually explored. One such future application is the adaptation of the robotic endoscopy for the performance of NOTES.^19^

NOTES is a minimally invasive surgery where the surgeon operates a flexible endoscope to access the intraabdominal cavity through transoral, transcolonic, or transvaginal routes. Because of its “scarless” surgical approach, patients frequently report lower post‐operative pain, shorter hospital stay, faster return to work, and improved cosmetic outcome.^20^ Nevertheless, performing NOTES is technically demanding because of the lack of triangulation provided by existing endoscopic equipment and the difficulty in securing the inner entry point created during NOTES. As suggested by the ASGE/SAGES Working Group on NOTES, the development of a novel operating platform addressing the limitations of triangulation is the crux for reviving NOTES.^21^

Because of its unique features of triangulation, flexible tool manipulation, and end‐effector telemanipulation, the Endomaster EASE System lends itself as an ideal operating platform to overcome the existing problems faced in performing NOTES.^22^ Nevertheless, the existing features of the Endomaster EASE System does not allow for effective endoscopic suturing which is essential in advancing the field of NOTES. We have hence developed a new capability of the Endomaster EASE System, allowing it to perform effective suturing and knot tying.^23^ Animal studies have been performed, which successfully demonstrated the feasibility of suturing and knot tying using this new capability.^7^

Atallah *et al*. demonstrated that the next generation robotic platforms can be used to perform a robotic trans‐cecal NOTES appendicectomy and a transvaginal unilateral salphingo‐oophorectomy with transvaginal extraction of the ovary and fallopian tube without requiring laparoscopic assistance.^24^ We believe that with the existing triangulation features of the Endomaster EASE System and the added‐on benefit of the novel suturing device, the Endomaster EASE System would be able to perform NOTES effectively. Our team has hence embarked on animal trials to evaluate the feasibility of using the Endomaster EASE System to perform NOTES appendicectomy.

## Conclusion

The successful adoption of the master–slave robotic concept has led to the development of numerous robotic endoscopic platforms. Because of the high degrees of freedom of the robotic arms, challenges encountered performing complex therapeutic procedures with conventional endoscopes can now be overcome. With the robotic endoscope, procedurists can perform complex therapeutic procedures with reduced difficulty and potentially lower complication rates. Nevertheless, a successful robotic endoscope must provide beyond the basic requirement of a high functioning endoscopic resection tool. It must be demonstrated to be safe, and cost‐effective for its intended clinical application. Training can be best achieved through the synergy of artificial‐intelligence‐guided deep learning systems and VR. NOTES is an ideal choice for the application of the robotic endoscope as the triangulation afforded by the device effectively solves the challenges faced when performing NOTES using existing endoscopic equipment.

## References

[1] Klibansky D , Rothstein RI . Robotics in endoscopy. Curr. Opin. Gastroenterol. 2012; 28: 477–82. 22885946 10.1097/MOG.0b013e328356ac5e

[2] Khan R , Plahouras J , Johnston BC , Scaffidi MA , Grover SC , Walsh CM . Virtual reality simulation training in endoscopy: a cochrane review and meta‐analysis. Endoscopy. 2019; 51: 653–64. 31071757 10.1055/a-0894-4400

[3] Bric JD , Lumbard DC , Frelich MJ , Gould JC . Current state of virtual reality simulation in robotic surgery training: a review. Surg. Endosc. 2016; 30: 2169–78. 26304107 10.1007/s00464-015-4517-y

[4] Visconti TAC , Otoch JP , Artifon ELA . Robotic endoscopy. A review of the literature. Acta Cir. Bras. 2020; 35: e202000206. 32348403 10.1590/s0102-865020200020000006 PMC7184939

[5] Lane T . A short history of robotic surgery. Ann. R. Coll. Surg. Engl. 2018; 100 (6_sup): 5–7. 29717892 10.1308/rcsann.supp1.5 PMC5956578

[6] Kaan HL , Ho KY . Robot‐assisted endoscopic resection: current status and future directions. Gut Liver. 2020; 14: 150–2. 31158954 10.5009/gnl19047 PMC7096234

[7] Cao L , Li XG , Phan PT , Kaan HL *et al* Sewing up the wounds: a robotic suturing system for flexible endoscopy. IEEE Robot. Autom. Mag. 2020 10.1109/MRA.2019.2963161.

[8] Zorn L , Nageotte F , Zanne P *et al* A novel telemanipulated robotic assistant for surgical endoscopy: preclinical application to ESD. IEEE Trans. Biomed. Eng. 2018; 65: 797–808. 28678698 10.1109/TBME.2017.2720739

[9] Kaan HL , Ho KY . Endoscopic robotic suturing: the way forward. Saudi J. Gastroenterol. 2019; 25: 272–6. 30900610 10.4103/sjg.SJG_12_19 PMC6784431

[10] Phee SJ , Reddy N , Chiu PW *et al* Robot‐assisted endoscopic submucosal dissection is effective in treating patients with early‐stage gastric neoplasia. Clin. Gastroenterol. Hepatol. 2012; 10: 1117–21. 22642951 10.1016/j.cgh.2012.05.019

[11] Fujimoto A , Goto O , Nishizawa T *et al* Gastric ESD may be useful as accurate staging and decision of future therapeutic strategy. Endosc. Int. Open. 2017; 5: E90–5. 28210705 10.1055/s-0042-119392 PMC5303017

[12] Turiani Hourneaux de Moura D , Aihara H , Jirapinyo P *et al* Robot‐assisted endoscopic submucosal dissection versus conventional ESD for colorectal lesions: outcomes of a randomized pilot study in endoscopists without prior ESD experience (with video). Gastrointest. Endosc. 2019; 90: 290–8. 30922861 10.1016/j.gie.2019.03.016

[13] Wang Z , Phee SJ , Lomanto D *et al* Endoscopic submucosal dissection of gastric lesions by using a master and slave transluminal endoscopic robot: an animal survival study. Endoscopy. 2012; 44: 690–4. 22723184 10.1055/s-0032-1309404

[14] Ho KY , Phee SJ , Shabbir A *et al* Endoscopic submucosal dissection of gastric lesions by using a Master and Slave Transluminal Endoscopic Robot (MASTER). Gastrointest. Endosc. 2012; 72: 593–5. 10.1016/j.gie.2010.04.009 20646698

[15] Chiu PW , Phee SJ , Bhandari P *et al* Enhancing proficiency in performing endoscopic submucosal dissection (ESD) by using a prototype robotic endoscope. Endosc. Int. Open. 2015; 3: E439–42. 26528498 10.1055/s-0034-1393178 PMC4612240

[16] de Lange T , Halvorsen P , Riegler M . Methodology to develop machine learning algorithms to improve performance in gastrointestinal endoscopy. World J. Gastroenterol. 2018; 24: 5057–62. 30568383 10.3748/wjg.v24.i45.5057 PMC6288655

[17] Hirasawa T , Aoyama K , Tanimoto T *et al* Application of artificial intelligence using a convolutional neural network for detecting gastric cancer in endoscopic images. Gastric Cancer. 2018; 21: 653–60. 29335825 10.1007/s10120-018-0793-2

[18] Everson M , Herrera L , Li W *et al* Artificial intelligence for the real‐time classification of intrapapillary capillary loop patterns in the endoscopic diagnosis of early oesophageal squamous cell carcinoma: a proof‐of‐concept study. United European Gastroenterol. J. 2019; 7: 297–306. 10.1177/2050640618821800 PMC6498793 31080614

[19] Zuo S , Wang S . Current and emerging robotic assisted intervention for NOTES. Expert Rev. Med. Devices. 2016; 13: 1095–105. 27788609 10.1080/17434440.2016.1254037

[20] Pearl JP , Ponsky JL . Natural orifice transluminal endoscopic surgery: a critical review. J. Gastrointest. Surg. 2008; 12: 1293–300. 18057995 10.1007/s11605-007-0424-4

[21] Rattner D , Kalloo A . ASGE/SAGES working group on natural orifice translumenal endoscopic surgery. Surg. Endosc. 2006; 20: 329–33. 16402290 10.1007/s00464-005-3006-0

[22] Phee SJ , Low SC , Huynh VA *et al* Master and Slave Transluminal Endoscopic Robot (MASTER) for Natural Orifice Transluminal Endoscopic Surgery (NOTES). Conf. Proc. IEEE Eng. Med. Boil. Soc. 2009; 2009: 1192–995. 10.1109/IEMBS.2009.5333413 19963992

[23] Kaan HL , Ho KY . Endoscopic full thickness resection for gastrointestinal tumors – challenges and solutions. Clin. Endosc. 2020 10.5946/ce.2019.161. PMC7548150 32061203

[24] Atallah S , Hodges A , Larach SW . Direct target NOTES: prospective applications for next generation robotic platforms. Tech. Coloproctol. 2018; 22: 363–71. 29855814 10.1007/s10151-018-1788-z

